# Association Between Area‐Level Socioeconomic Disadvantage and Immunotherapy in Patients With Non‐Small Cell Lung Cancer

**DOI:** 10.1002/cam4.71038

**Published:** 2025-07-10

**Authors:** Hiroe Suzuki‐Chiba, Atsushi Miyawaki, Taiki Hakozaki, Shotaro Aso, Hiroki Matsui, Hideo Yasunaga

**Affiliations:** ^1^ Department of Health Communication Graduate School of Medicine, The University of Tokyo Tokyo Japan; ^2^ Department of Health Services Research Graduate School of Medicine, The University of Tokyo Tokyo Japan; ^3^ Department of Clinical Epidemiology and Health Economics School of Public Health, Graduate School of Medicine, The University of Tokyo Tokyo Japan; ^4^ Department of Thoracic Oncology and Respiratory Medicine Tokyo Metropolitan Cancer and Infectious Diseases Center, Komagome Hospital Tokyo Japan

**Keywords:** health disparities, immunotherapy, non‐small cell lung cancer, socioeconomic status

## Abstract

**Background:**

Recent advances in immunotherapy have improved the survival of patients with non‐small cell lung cancer (NSCLC). However, data on the association between patient socioeconomic status (SES) and the use of immunotherapy remain scarce. We examined the association between area‐level SES and immunotherapy use in patients with stage IV NSCLC in Japan.

**Methods:**

Data from a national inpatient database were used for this analysis. Patients aged ≥ 18 years, hospitalized for stage IV NSCLC, and treated with immunotherapy alone, immunotherapy combined with platinum‐based chemotherapy, and platinum‐based chemotherapy alone as first‐line pharmacotherapy from April 2016 to March 2022 were analyzed. Area‐level SES was measured using the area deprivation index and categorized into quartiles. The primary outcome was the use of immunotherapy as a first‐line treatment. A multivariate linear regression model was used, adjusted for patient characteristics, years, and hospital fixed effects.

**Results:**

A total of 47,291 eligible patients from 843 hospitals, with 22,205 (47%) receiving immunotherapy. Adjusted analyses showed that patients in the most disadvantaged area were less likely to receive immunotherapy compared with those in the least disadvantaged area (adjusted difference, −2.0 percentage points [pp]; 95% confidence interval, −3.6 to −0.5 pp; *p* = 0.01). Although not significant, this trend persisted when stratified by urban and cancer‐designated hospitals. However, this difference was no longer significant after adjusting for hospital fixed effects.

**Conclusion:**

In Japan, with universal public health insurance with low out‐of‐pocket costs, living in a socioeconomically disadvantaged area is associated with lower rates of immunotherapy use among patients with stage IV NSCLC. These disparities disappeared within the same hospitals using hospital fixed effects; however, such disparities tended to persist in urban, non‐academic, and cancer‐designated hospitals. These findings suggest the need for interventions to address the structural barriers, including those within the hospitals, to optimize NSCLC treatment and reduce health disparities.

## 1 Introduction

Social determinants of health play a crucial role in cancer outcomes, with both individual and area‐level socioeconomic status (SES) emerging as significant predictors of survival rates across various cancer types [1, 2, 3, 4]. Non‐small cell lung cancer (NSCLC) demonstrates a strong association with SES as both its incidence and treatment outcomes are closely associated with income, educational levels, occupational status, social isolation, and healthcare access [5, 6, 7, 8]. The recent advent of immunotherapy, both as monotherapy and in combination with platinum‐based chemotherapy, has improved survival outcomes for patients with NSCLC [9, 10]. However, despite these therapeutic advances, socioeconomic disparities persist in treatment access. High medication costs and the complexity of managing adverse events continue to create barriers for patients and caregivers from lower socioeconomic backgrounds [11, 12, 13, 14].

Area‐level SES has received particular attention, as it can affect cancer outcomes in patients with NSCLC through various pathways, including access to hospital or healthcare resources, availability of caregivers, and levels of social isolation [4, 8, 14, 15]. Nevertheless, evidence on the association between area‐level SES and the receipt of immunotherapy remains limited. Previous studies have identified disparities in immunotherapy receipt according to area‐level SES; however, most studies were conducted in countries lacking universal healthcare systems [16, 17]. Recently, similar patterns were observed in other developed countries with universal healthcare systems [18].

However, the generalizability of these findings to other countries remains limited as the availability of immunotherapy has been affected by variations in health insurance coverage and the quality of hospital care across countries [19]. Whether socioeconomic disadvantages are associated with receipt of immunotherapy in a setting such as Japan—where a standardized public health insurance system and low out‐of‐pocket costs based on household income are implemented—has yet to be determined.

Therefore, using a nationwide inpatient database in Japan, where a standardized public health insurance system minimizes the effect of economic barriers [20, 21], we aimed to investigate whether area‐level socioeconomic disadvantages were associated with the receipt of immunotherapy, after adjusting for patient characteristics and hospital fixed effects. Socioeconomic disadvantages were assessed using the area deprivation index (ADI) validated by previous studies [22].

## 2 Methods

### 2.1 Data Source

This observational study used the Diagnosis Procedure Combination database, a nationwide inpatient database in Japan [23]. This database contained hospital administrative claims data from > 1000 acute care hospitals, encompassing approximately 8 million inpatient admissions annually. Participation in this database is mandatory for academic hospitals and voluntary for community hospitals. The database on inpatient care contains information on patients' age and sex, residential zip code, body height and weight, smoking history, tumor‐node‐metastasis stage of malignant tumors at admission, primary diagnosis, comorbidities identified at admission, complications occurring after admission (recorded using the *International Classification of Diseases, 10th Revision* [ICD‐10] codes), discharge status, activities of daily living at admission and discharge, medical procedures and medications administered during hospitalization, and unique identifiers of the hospital. A previous validation study reported a specificity of 96.7% for recorded diagnoses of lung cancer and a sensitivity of 50%–80% [24]. Both the specificity and sensitivity of the recorded procedures exceeded 90% [25].

Hospital information was obtained from the Annual Report for Functions of Medical Institutions, which has provided detailed hospital information and statistics since 2014 [26]. These two databases were linked using the unique hospital identifier.

This study was approved by the Institutional Review Board of the University of Tokyo (approval number: 3501‐5; May 19, 2021). The requirement for informed consent was waived as de‐identified data were used prior to the analysis.

### 2.2 Patient Population

Patients aged ≥ 18 years who were hospitalized with a diagnosis of NSCLC (ICD‐10 code: C34) and received first‐line pharmacotherapy for the first time between April 2016 and March 2022 were analyzed. NSCLC was identified using Japanese text data [27]. The first‐line pharmacotherapy for NSCLC included the following treatments, all of which are standard for stage IV NSCLC without targetable mutation in Japan [28, 29]: (i) immunotherapy, with or without concomitant cisplatin‐ or carboplatin‐based chemotherapy, and (ii) cisplatin‐ or carboplatin‐based chemotherapy alone. Immunotherapy was defined as treatment with nivolumab, pembrolizumab, atezolizumab, or durvalumab. Patients diagnosed with stage I–III NSCLC and treated with a combination of pharmacotherapy, etoposide, and irinotecan were excluded. This selection criterion aimed to enhance the accuracy of NSCLC diagnosis in Japan [28]. Finally, patients whose residential SES and rurality were not identifiable were also excluded.

### 2.3 Exposure Variable

The exposure variable was the patients' residential area‐level ADI (categorized into quartiles at the zip code level as an indicator of area‐level SES) [22]. The ADI was developed in a previous study using a method similar to the Breadline Britain poverty measure and the European transnational ecological deprivation measure [30, 31]. It is a composite indicator of geographical SES by zip code, defined as the weighted sum of eight poverty‐related census variables. The standardized ADI was used, which represents the relative position of each zip code within the entire population of Japan, ranging from zero (least socioeconomically disadvantaged) to one (most socioeconomically disadvantaged). The components and formulas used to calculate the ADI are provided in the Appendix S1. The standardized ADI was divided into quartiles at the zip code area level, with the highest ADI value (quartile 4) representing the most socioeconomically disadvantaged. This information was matched with the quartiles of the standardized ADI and the patients' residences at the zip code level, reflecting nationwide patterns of socioeconomic disadvantage as shown in the previous study [32].

### 2.4 Outcomes

The primary outcome measure was the receipt of immunotherapy. In this study, immunotherapy was defined as the administration of immunotherapy, with or without concomitant cisplatin‐ or carboplatin‐based chemotherapy.

### 2.5 Adjustment Variables

Adjustments were made for both patient and hospital characteristics. Patient characteristics included sex, age, body mass index, smoking index (0, 1–19, or ≥ 20 pack‐years), baseline comorbidities assessed using the Charlson Comorbidity Index [33], interstitial lung disease [34], connective tissue disease [35], activities of daily living on admission, an indicator of rurality based on the patient's residential area [36], emergency admission status, and fiscal year of admission. Hospital characteristics included hospital size (large [≥ 400 beds], medium [100–399], and small [≤ 99]), teaching status (academic or non‐academic hospital), and the rurality index of the hospital (1–10 [urban] or 11–100 [rural]).

Age was categorized into four groups: 18–64, 65–74, 75–84, and ≥ 85 years. Body mass index was classified into < 18.5, 18.5–21.9, 22.0–24.9, 25.0–29.9, and ≥ 30.0 kg/m^2^. The Charlson Comorbidity Index was determined using ICD‐10 codes and categorized as 2, 3, 4, or 5. As all eligible patients were diagnosed with NSCLC, their Charlson Comorbidity Index score was ≥ 2. Additionally, interstitial lung disease (ICD‐10 codes: B22.1, J70.0–J70.4, J84.1, J84.9, and J99) [37] and connective tissue disease (M05, M06, and M30–M36). Activities of daily living on admission were evaluated using the Barthel Index (0–100) [38]. The patients were divided into four groups based on their Barthel Index: 0–40 (total or very independent), 41–80 (partially or minimally independent), 81–100 (independent), and missing data [39]. Patients' residential areas were evaluated using the rurality index for Japan [36], an indicator of rurality matched by zip codes. The rurality index for Japan comprises four factors: population density, distance to secondary or tertiary care hospitals, remote islands, and heavy snow areas. This index ranges from 1 (most urban) to 100 (most rural); its validity was confirmed in a previous validation study [36].

### 2.6 Statistical Analyses

First, the overall frequencies (%) and means (standard deviations) of baseline patient and hospital characteristics were compared across ADI quartiles. Continuous and categorical variables were compared using the *t*‐test and chi‐squared test, respectively.

Second, the association between the receipt of immunotherapy and area‐level socioeconomic disadvantages was examined using multivariate logistic regression models, with standard errors clustered at the hospital level to account for potential intra‐hospital correlation. The adjusted rates of immunotherapy receipt were calculated according to the ADI quartiles. To enhance the interpretability of the findings, the average marginal effects for each ADI group, relative to quartile 1 (the least socioeconomically disadvantaged), were calculated instead of odds ratios. These average marginal effects represent the differences in the adjusted probabilities of receiving immunotherapy across ADI groups. Model 1 was adjusted for patient‐level covariates, as described in the previous section. Model 2 included hospital fixed effects (with hospital identifiers used as adjustment variables) to account for the influence of measured and unmeasured time‐invariant hospital characteristics on the findings. Individual hospital characteristics were not adjusted separately, as the inclusion of hospital fixed effects accounted for all time‐invariant and unmeasured hospital‐level characteristics.

Third, additional stratified analyses were conducted based on hospital type (urban or rural) [36], teaching status, cancer‐designated status [40], and hospital size.

Finally, using the same regression model as in Model 1, the overall trends in the receipt of immunotherapy from 2016 to 2022 were examined across ADI quartiles. Interaction terms between ADI quartile indicators and fiscal year indicators were incorporated into the model and tested to assess whether temporal differences in immunotherapy receipt varied across ADI quartiles throughout the study period.

All statistical analyses were performed using the Stata/SE 17.0 software (StataCorp, College Station, TX, USA). All tests were two‐sided, with significance defined as a *p* value of < 0.05 or assessed based on 95% confidence intervals (CIs).

### 2.7 Sensitivity Analyses

Two sensitivity analyses were conducted. First, the analysis was repeated after matching the standardized ADI to the residential areas of patients with stage IV NSCLC, ensuring equal distribution of patients across each ADI quartile. This approach aimed to minimize the loss of statistical power. Second, the analysis was repeated using ADI deciles. A range was created, with the least socioeconomically disadvantaged in the first decile and the most socioeconomically disadvantaged in the tenth decile.

## 3 Results

### 3.1 Study Population and Patient and Hospital Characteristics

**FIGURE 1:**
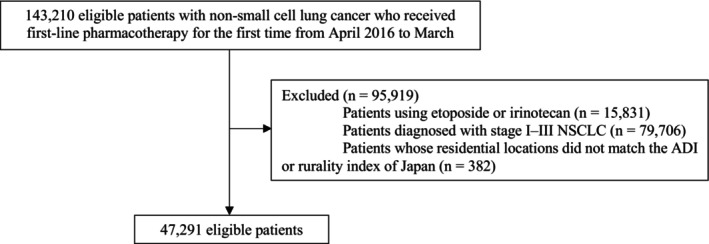
Flowchart of the patient selection process.

A total of 143,210 patients with NSCLC (aged ≥ 18 years) treated with first‐line pharmacotherapy for the first time were identified. Of these, 15,831 patients treated with pharmacotherapy combined with etoposide or irinotecan, 79,706 with stage I–III NSCLC, and 382 whose residential locations did not match the ADI or rurality index for Japan were excluded. Ultimately, 47,291 patients were eligible for analysis (Figure 1). The mean (standard deviation) age of these individuals was 68.3 (9.1) years, and 11,763 (24.9%) were women. Among them, 22,205 (47.3%) received immunotherapy. Overall, 27,538 (58.2%) patients lived in the quartile of zip code areas with the least socioeconomically disadvantaged (quartile 1), whereas 6398 (13.5%) patients lived in the quartile of zip code areas with the most socioeconomically disadvantaged (quartile 4) (Table 1). Patients who lived in more socioeconomically disadvantaged areas (i.e., those in higher ADI quartiles) were less likely to be young, more likely to be current or past smokers, more likely to have comorbidities, and more likely to live in rural areas compared with those in less socioeconomically disadvantaged areas. Furthermore, these patients were less likely to be treated in large hospitals, academic hospitals, and cancer‐designated hospitals (Table 1).

**TABLE 1:** Characteristics of patients based on ADI quartiles.

	All patients	ADI quartiles				*p*
		Quartile 1 (least disadvantage)	Quartile 2	Quartile 3	Quartile 4 (most disadvantage)	
No. of patients	47,291	27,538	6650	6705	6398	
Male, *n* (%)	35,528 (75.1)	20,692 (75.1)	5048 (75.9)	5055 (75.4)	4733 (74.0)	0.07
Age, years, *n* (%)						0.00
18–64	12,965 (27.4)	7729 (28.1)	1857 (27.9)	1777 (26.5)	1602 (25.0)	
65–74	22,510 (47.6)	12,922 (46.9)	3155 (47.4)	3229 (48.2)	3134 (49.0)	
75–84	11,209 (23.7)	6522 (23.7)	1565 (23.5)	1548 (23.1)	1574 (24.6)	
≥ 85	607 (1.3)	365 (1.3)	73 (1.1)	81 (1.2)	88 (1.4)	
Body mass index, kg/m^2^, *n* (%)						0.04
< 18.5	7544 (16.0)	4305 (15.6)	1073 (16.1)	1106 (16.5)	1060 (16.6)	
18.5–21.9	17,629 (37.3)	10,333 (37.5)	2461 (37.0)	2455 (36.6)	2380 (37.2)	
22.0–24.9	13,921 (29.4)	8132 (29.5)	2008 (30.2)	1983 (29.6)	1798 (28.1)	
25.0–29.9	7090 (15.0)	4160 (15.1)	952 (14.3)	992 (14.8)	986 (15.4)	
≥ 30.0	−825 (1.7)	440 (1.6)	123 (1.8)	132 (2.0)	130 (2.0)	
Missing data	282 (0.6)	168 (0.6)	33 (0.5)	37 (0.6)	44 (0.7)	
Smoking index pack‐years, *n* (%)						< 0.001
0	11,479 (24.3)	6982 (25.4)	1538 (23.1)	1553 (23.2)	1406 (22.0)	
1–19	3886 (8.2)	2349 (8.5)	501 (7.5)	522 (7.8)	514 (8.0)	
≥ 20	28,288 (59.8)	15,987 (58.1)	4119 (61.9)	4155 (62.0)	4027 (62.9)	
Missing data	3638 (7.7)	2220 (8.1)	492 (7.4)	475 (7.1)	451 (7.0)	
Charlson Comorbidity Index score, *n* (%)						< 0.001
2	20,857 (44.1)	12,542 (45.5)	2830 (42.6)	2860 (42.7)	2625 (41.0)	
3	4042 (8.5)	2344 (8.5)	596 (9.0)	566 (8.4)	536 (8.4)	
4	1268 (2.7)	695 (2.5)	173 (2.6)	181 (2.7)	219 (3.4)	
≥ 5	21,115 (44.6)	11,952 (43.4)	3049 (45.8)	3097 (46.2)	3017 (47.2)	
Barthel Index score, *n* (%)						0.16
1 (0–40)	654 (1.4)	352 (1.3)	101 (1.5)	106 (1.6)	95 (1.5)	
2 (41–80)	2794 (5.9)	1565 (5.7)	429 (6.5)	406 (6.1)	394 (6.2)	
3 (81–100)	41,483 (87.7)	24,242 (88.0)	5785 (87.0)	5863 (87.4)	5593 (87.4)	
Missing data	2360 (5.0)	1379 (5.0)	335 (5.0)	330 (4.9)	316 (4.9)	
Interstitial lung disease, *n* (%)	2075 (4.4)	1203 (4.4)	267 (4.0)	319 (4.8)	286 (4.5)	0.40
Collagen disease, *n* (%)	868 (1.8)	500 (1.8)	117 (1.8)	135 (2.0)	116 (1.8)	0.21
Emergency admission, *n* (%)	1020 (2.2)	561 (2.0)	160 (2.4)	153 (2.3)	146 (2.3)	0.41
Rural index, mean (standard deviation)	31 (25.5)	28 (23.3)	35 (25.2)	36 (26.3)	36 (29.9)	< 0.01
Fiscal year of pharmacotherapy, *n* (%)						0.29
2016	8463 (17.0)	4904 (17.8)	1208 (18.2)	1198 (17.9)	1153 (18.0)	
2017	7659 (16.2)	4417 (16.0)	1091 (16.4)	1099 (16.4)	1052 (16.4)	
2018	7920 (16.7)	4519 (16.4)	1139 (17.1)	1152 (17.2)	1110 (17.3)	
2019	7706 (16.3)	4573 (16.6)	1073 (16.1)	1070 (16.0)	990 (15.5)	
2020	8067 (17.1)	4661 (16.9)	1135 (17.1)	1156 (17.2)	1115 (17.4)	
2021	7476 (15.8)	4464 (16.2)	1004 (15.1)	1030 (15.4)	978 (15.3)	
Hospital characteristics for pharmacotherapy						
Hospital size, *n* (%)						< 0.01
Large (≥ 400 beds)	33,184 (70.2)	20,450 (74.3)	4470 (67.2)	4304 (64.2)	3960 (61.9)	
Medium (100–399 beds)	9738 (20.6)	4898 (17.8)	1541 (23.2)	1658 (24.7)	1641 (25.6)	
Small (≤ 99 beds)	601 (1.3)	282 (1.0)	83 (1.2)	122 (1.8)	114 (1.8)	
Missing data	3768 (8.0)	1908 (6.9)	556 (8.4)	621 (9.3)	683 (10.7)	
Academic hospital, *n* (%)	26,873 (56.8)	15,588 (56.6)	3884 (58.4)	3818 (56.9)	3583 (56.0)	< 0.01
Cancer‐designated hospital, *n* (%)	19,359 (40.9)	11,513 (41.8)	2754 (41.4)	2669 (39.8)	2423 (37.9)	< 0.01

Abbreviation: ADI, area deprivation index.

The distribution of hospitals where patients received pharmacotherapy was uneven across ADI quartiles, as shown in Table 2. Patients who lived in more socioeconomically disadvantaged areas were less likely to receive treatment in academic hospitals compared with those who lived in less socioeconomically disadvantaged areas.

**TABLE 2:** Characteristics of hospitals (*n* = 832) based on ADI quartiles.

Characteristics	ADI quartile, no. (%)			
	Quartile 1 (least disadvantage)	Quartile 2	Quartile 3	Quartile 4 (most disadvantage)
No. of hospitals^a^, *n* (%)	780	661	656	622
Hospitals' rurality index, *n* (%)				
1–10 (urban)	144 (18.5)	113 (17.1)	111 (16.9)	97 (15.6)
11–100 (rural)	556 (71.3)	477 (72.2)	473 (72.1)	473 (76.0)
Missing	80 (10.3)	72 (10.9)	72 (11.0)	72 (11.6)
Hospital size, *n* (%)				
Large (≥ 400 beds)	352 (45.1)	337 (51.0)	332 (50.6)	300 (49.7)
Medium (100–399 beds)	336 (43.1)	246 (37.2)	246 (37.5)	239 (38.4)
Small (≤ 99 beds)	14 (1.8)	9 (1.4)	10 (1.5)	9 (1.4)
Missing data	78 (10.0)	62 (9.4)	68 (10.4)	65 (10.5)
No. of academic hospital, *n* (%)	685 (87.8)	562 (85.0)	558 (85.1)	514 (82.6)
No. of cancer‐designated hospital, *n* (%)	215 (27.6)	209 (31.6)	205 (31.3)	195 (31.4)

Abbreviation: ADI, area deprivation index.

^a^ A total of 832 hospitals were included in the analysis.

### 3.2 Receipt of Immunotherapy by Area‐Level Socioeconomic Status

**FIGURE 2:**
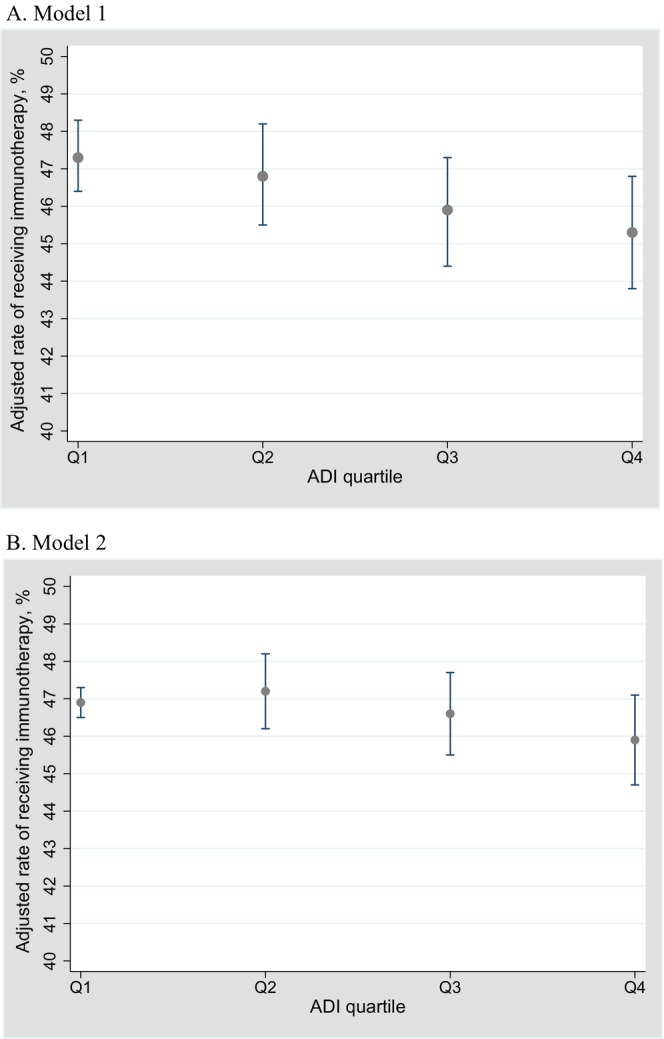
Adjusted rates of immunotherapy receipt stratified based on ADI quartiles of (A) Model 1 and (B) Model 2.

After adjusting for patient characteristics in Model 1, the rate of immunotherapy significantly declined from the quartile 1 areas (adjusted receiving immunotherapy rate, 47.3% [95% CI, 46.4–48.3]) to the quartile 4 areas (45.3% [95% CI, 43.8–46.8]; average marginal effects for areas vs. quartile 4 areas, −2.0 percentage points [95% CI, −3.6 to −0.5 percentage points]; *p* < 0.01) (Figure 2; Table 3). In Model 2, after adjusting for patient characteristics and hospital fixed effects, no significant association was observed between the use of immunotherapy and socioeconomic disadvantages. The adjusted immunotherapy receipt rate slightly decreased from the quartile 1 areas (adjusted immunotherapy receipt rate, 46.9% [95% CI, 46.5–47.3]) to the quartile 4 areas (45.9% [95% CI, 44.7 to 47.1]; average marginal effects for the quartile 1 areas vs. quartile 4 areas, −1.1 percentage points [95% CI, −2.5 to 0.4 percentage points]; *p* = 0.15) (Figure 2; Table 3).

**TABLE 3:** Association between the use of immunotherapy as first‐line treatment and ADI quartiles.

ADI quartile	No. of patients	Model 1			Model 2		
		Adjusted rate, % (95% CI)	Average marginal effect, % (95% CI)	*p*	Adjusted rate, % (95% CI)	Average marginal effect, % (95% CI)	*p*
Quartile 1 (least disadvantage)	27,538	47.3 (46.4–48.3)	Reference		46.9 (46.5–47.3)	Reference	
Quartile 2	6650	46.8 (45.5–48.2)	−0.5 (−1.8 to 0.9)	0.47	47.2 (46.2–48.2)	−0.2 (−0.9 to 1.5)	0.67
Quartile 3	6705	45.9 (44.4–47.3)	−1.4 (−2.9 to −0.02)	0.05	46.6 (45.5–47.7)	−0.4 (−1.7 to 1.0)	0.60
Quartile 4 (most disadvantage)	6398	45.3 (43.8–46.8)	−2.0 (−3.6 to −0.5)	0.01	45.9 (44.7–47.1)	−1.1 (−2.5 to 0.4)	0.15

*Note:* Adjustments were made for sex, age, body mass index, smoking index, Charlson Comorbidity Index score, interstitial lung disease, connective tissue disease, Barthel Index score, and rural index matched by patient residential area, emergency admission, and fiscal year of admission (Model 1). To account for potential correlation among patients treated at the same hospital, hospital fixed effects were additionally included in the model (Model 2).

Abbreviations: ADI, area deprivation index; CI, confidence interval.

### 3.3 Receipt of Immunotherapy by Area‐Level Socioeconomic Status Stratified by Hospital Type

In the analysis stratified by urban–rural hospital classification (Table 4), the use of immunotherapy was consistently lower in urban hospitals compared with rural hospitals. Although not significant, the rate of immunotherapy in urban hospitals showed a tendency to decline from quartile 1 areas (adjusted immunotherapy receipt rate, 45.6% [95% CI, 43.7–47.5]) to the quartile 4 areas (42.5% [95% CI, 38.3–46.8]; average marginal effects for areas vs. quartile 4 areas, −3.1 percentage points [95% CI, −7.3 to 1.1 percentage points]; *p* = 0.091). When stratified by hospital teaching status (Table 5), immunotherapy use was consistently lower in academic hospitals compared with non‐academic hospitals. In non‐academic hospitals, the use of immunotherapy significantly declined from the quartile 1 areas (adjusted immunotherapy receipt rate, 49.1% [95% CI, 47.9–50.3]) to the quartile 4 areas (46.1% [95% CI, 44.0–48.2]; average marginal effects for areas vs. quartile 4 areas, −3.0 percentage points [95% CI, −5.1 to −0.8 percentage points]; *p* = 0.006). Although not significant, when stratified by cancer‐designated hospitals (Table 6), the use of immunotherapy in non‐cancer‐designated hospitals tended to decrease from the quartile 1 areas (adjusted immunotherapy receipt rate, 46.3% [95% CI, 45.0–47.7]) to the quartile 4 areas (44.1% [95% CI, 41.8–46.3]; average marginal effects for areas vs. quartile 4 areas, −2.3 percentage points [95% CI, −4.6 to 0.01 percentage points]; *p* = 0.064). In the analysis stratified by hospital size (Table 7), medium‐sized hospitals showed a significant decline in immunotherapy use from the quartile 1 areas (adjusted immunotherapy receipt rate, 47.3% [95% CI, 45.4–49.3]) to the quartile 2 areas (44.1% [95% CI, 41.7–46.5]) and the quartile 3 areas (43.8% [95% CI, 40.9–46.7]; average marginal effects for areas vs. the quartile 2 areas, −3.2 percentage points ([95% CI, −5.6 to −0.8 percentage points]; *p* = 0.009) and average marginal effects for areas versus the quartile 3 areas, −3.6 percentage points [95% CI, −6.3 to −0.8 percentage points]; *p* = 0.012).

**TABLE 4:** Association between the use of immunotherapy as first‐line treatment and ADI quartiles stratified by urban–rural hospital classification.

ADI quartile	No. of patients	Model 1					
		Urban hospital			Rural hospital		
		Adjusted rate, % (95% CI)	Average marginal effect, % (95% CI)	*p*	Adjusted rate, % (95% CI)	Average marginal effect, % (95% CI)	*p*
Quartile 1 (least disadvantage)	27,538	45.6 (43.7–47.5)	Reference		46.9 (45.8–48.1)	Reference	
Quartile 2	6650	44.1 (40.7–47.5)	−1.5 (−4.8 to 1.8)	0.36	46.7 (45.2–48.3)	−0.2 (−1.8 to 1.3)	0.77
Quartile 3	6705	42.1 (38.3–46.0)	−3.5 (−7.1 to 0.2)	0.064	46.2 (44.7–47.8)	−0.7 (−2.3 to 0.8)	0.37
Quartile 4 (most disadvantage)	6398	42.5 (38.3–46.8)	−3.1 (−7.3 to 1.1)	0.091	45.8 (44.1–47.5)	−1.1 (−3.0 to 0.7)	0.21

*Note:* Adjustments were made for sex, age, body mass index, smoking index, Charlson Comorbidity Index score, interstitial lung disease, connective tissue disease, Barthel Index score, and rural index matched by patient residential area, emergency admission, and fiscal year of admission (Model 1).

Abbreviations: ADI, area deprivation index; CI, confidence interval.

**TABLE 5:** Association between the use of immunotherapy as first‐line treatment and ADI quartiles stratified by academic and non‐academic hospital.

ADI quartile	No. of patients	Model 1					
		Academic hospital			Non‐academic hospital		
		Adjusted rate, % (95% CI)	Average marginal effect, % (95% CI)	*p*	Adjusted rate, % (95% CI)	Average marginal effect, % (95% CI)	*p*
Quartile 1 (least disadvantage)	27,538	45.9 (44.8–47.1)	Reference		49.1 (47.9–50.3)	Reference	
Quartile 2	6650	45.8 (44.1–47.5)	−0.2 (−1.8 to 1.5)	0.86	48.2 (46.3–50.2)	−0.8 (−2.8 to 1.2)	0.48
Quartile 3	6705	44.4 (42.5–46.2)	−1.6 (−3.5 to 0.3)	0.11	47.9 (45.8–49.9)	−1.1 (−3.1 to 0.7)	0.27
Quartile 4 (most disadvantage)	6398	44.7 (42.8–46.7)	−1.2 (−3.2 to 0.8)	0.24	46.1 (44.0–48.2)	−3.0 (−5.1 to −0.8)	0.006

*Note:* Adjustments were made for sex, age, body mass index, smoking index, Charlson Comorbidity Index score, interstitial lung disease, connective tissue disease, Barthel Index score, and rural index matched by patient residential area, emergency admission, and fiscal year of admission (Model 1).

Abbreviations: ADI, area deprivation index; CI, confidence interval.

**TABLE 6:** Association between the use of immunotherapy as first‐line treatment and ADI quartiles stratified by cancer‐designated and non‐cancer‐designated hospital.

ADI quartile	No. of patients	Model 1					
		Cancer‐designated hospital			Non‐cancer‐designated hospital		
		Adjusted rate, % (95% CI)	Average marginal effect, % (95% CI)	*p*	Adjusted rate, % (95% CI)	Average marginal effect, % (95% CI)	*p*
Quartile 1 (least disadvantage)	27,538	46.3 (45.0–47.7)	Reference		48.0 (46.7–49.3)	Reference	
Quartile 2	6650	47.1 (44.9–49.2)	0.7 (−1.4 to 2.9)	0.50	46.6 (44.9–48.3)	−1.4 (−3.1 to 0.2)	0.092
Quartile 3	6705	46.2 (44.1–48.3)	−0.1 (−2.2 to 2.0)	0.93	45.6 (43.6–47.6)	−2.4 (−4.3 to −0.5)	0.015
Quartile 4 (most disadvantage)	6398	44.1 (41.8–46.3)	−2.3 (−4.6 to 0.01)	0.064	46.2 (44.1–48.2)	−1.9 (−3.8 to 0.1)	0.068

*Note:* Adjustments were made for sex, age, body mass index, smoking index, Charlson Comorbidity Index score, interstitial lung disease, connective tissue disease, Barthel Index score, and rural index matched by patient residential area, emergency admission, and fiscal year of admission (Model 1).

Abbreviations: ADI, area deprivation index; CI, confidence interval.

**TABLE 7:** Association between the use of immunotherapy as first‐line treatment and ADI quartiles stratified by hospital size.

ADI quartile	No. of patients	Model 1								
		Large hospital (≥ 400 beds)			Medium hospital (100–399 beds)			Small hospital (≤ 99 beds)		
		Adjusted rate, % (95% CI)	Average marginal effect, % (95% CI)	*p*	Adjusted rate, % (95% CI)	Average marginal effect, % (95% CI)	*p*	Adjusted rate, % (95% CI)	Average marginal effect, % (95% CI)	*p*
Quartile 1	27,538	46.8 (45.6–47.9)	Reference		47.3 (45.4–49.3)	Reference		33.4 (26.2–40.6)	Reference	
Quartile 2	6650	47.3 (45.7–48.9)	0.5 (−1.2 to 2.2)	0.54	44.1 (41.7–46.5)	−3.2 (−5.6 to −0.8)	0.009	33.6 (23.3–43.8)	0.2 (−8.4 to 8.7)	0.97
Quartile 3	6705	46.6 (44.9–48.2)	−0.2 (−1.9 to 1.5)	0.81	43.8 (40.9–46.7)	−3.6 (−6.3 to −0.8)	0.012	30.4 (25.3–35.5)	−3.0 (−1.3 to 6.9)	0.53
Quartile 4	6398	45.3 (43.3–47.3)	−1.5 (−3.5 to 0.6)	0.16	45.1 (42.6–47.7)	−2.2 (−5.0 to 0.6)	0.13	33.9 (28.9–39.0)	0.5 (−5.0 to6.1)	0.85

*Note:* Adjustments were made for sex, age, body mass index, smoking index, Charlson Comorbidity Index score, interstitial lung disease, connective tissue disease, Barthel Index score, and rural index matched by patient residential area, emergency admission, and fiscal year of admission (Model 1).

Abbreviations: ADI, area deprivation index; CI, confidence interval.

### 3.4 Trends of Receiving Immunotherapy

**FIGURE 3:**
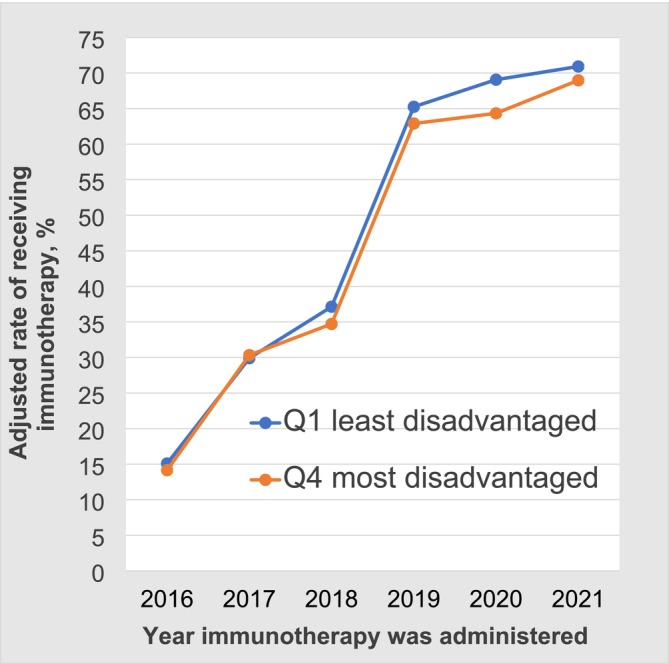
Trends in adjusted immunotherapy receipt rates for Q1 and Q4 in Model 1 from 2016 to 2021.

Overall, each ADI quartile showed an increasing trend in the adjusted immunotherapy rate in Model 1 from 2016 to 2021 (Figure S1). As shown in Figure 3, the adjusted immunotherapy receipt rate in the quartile 1 areas was consistently higher than that in the quartile 4 areas from 2018 to 2021. A significant difference was observed in 2020 between the quartile 1 areas (adjusted for immunotherapy receipt rate, 69.0% [95% CI, 67.7–70.3]) and the quartile 4 areas (adjusted for immunotherapy receipt rate, 64.3% [95% CI, 61.5–67.2]; *p* = 0.006).

### 3.5 Sensitivity Analyses

The findings remained qualitatively unchanged after dividing the samples into equal sizes based on ADI quartiles (Table S1). Additionally, the rate of immunotherapy in Model 1 by ADI deciles significantly declined from the decile 1 areas (adjusted immunotherapy receipt rate, 47.4%) to the decile 9 areas (45.0%; average marginal effects for decile 1 areas vs. the quartile 9 areas, −2.5 percentage points [95% CI, −4.5 to −0.5 percentage points]; *p* = 0.016) (Figure S2; Table S2).

## 4 Discussion

Using a national inpatient database in Japan, this observational study revealed socioeconomic disparities among patients receiving immunotherapy for stage IV NSCLC. After adjusting for patient and area characteristics, patients living in socioeconomically disadvantaged areas were less likely to receive immunotherapy compared with those living in socioeconomically advantaged areas. Although the difference was modest—approximately 2%—this disparity represents a significant number of patients. Over 100 patients from the most deprived areas may have been unable to access immunotherapy over the six‐year study period, given the inclusion of 6000 patients from these areas. Furthermore, the difference in immunotherapy receipt reached approximately 5% in 2020 (during the early stages of the coronavirus disease 2019 pandemic), making it clinically significant. Although the absolute difference appears small, immunotherapy has the potential to significantly improve prognosis in eligible patients with stage IV NSCLC cancer [9, 10]. Even modest disparities in its use could therefore lead to clinically meaningful differences, considering that more than 120,000 patients with lung cancer are diagnosed annually in Japan [41]. However, no significant difference was observed in outcomes when patients were treated at the same hospital. This finding suggests that the healthcare settings where patients with stage IV NSCLC receive initial treatment play a critical role in the administration of immunotherapy. Considering that immunotherapy is a new, costly, and complex treatment to administer and manage [13, 42], only certain hospitals appear to have the capacity to provide it to patients living in socioeconomically disadvantaged areas. This finding suggests the presence of structural or unmeasured barriers, other than patient‐level variables observed, which may persist even within a universal healthcare system and an out‐of‐pocket payment structure. To the best of our knowledge, this study is the first to examine the association between the receipt of immunotherapy and SES within the context of a standardized public health insurance system and low out‐of‐pocket payments for immunotherapy.

Disparities in immunotherapy use in Japan, despite universal health coverage and caps on out‐of‐pocket payments based on household income, remain unclear [20, 21]. This is especially surprising considering that only included patients began treatment with first‐line pharmacotherapy. Several potential mechanisms may explain these disparities. First, patients from socioeconomically disadvantaged areas often face barriers in accessing hospitals and receiving appropriate treatments, particularly when they live in rural areas. These barriers may include the need to drive cars or use public transportation, take time off work, and seek support from caregivers [3, 43]. Second, patients may be unaware of financial support schemes available to reduce out‐of‐pocket payments [11, 44]. In Japan, cancer counseling and support centers in hospitals provide information on reducing the financial and social burdens of treatment. However, the utilization rate of these services remains low [11, 45]. Thus, socioeconomically disadvantaged patients may lack access to information regarding ways to reduce their treatment burden, which may lead to the decision to forgo immunotherapy. Finally, patients from socioeconomically disadvantaged areas often experience challenges in obtaining information and understanding the importance of immunotherapy, as SES is closely associated with lower educational levels and health literacy [46, 47]. When healthcare providers proposed immunotherapy during initial discussions, most patients experienced difficulties in understanding the term and expressed anxiety about potential treatment outcomes [48, 49]. Consequently, patients from socioeconomically disadvantaged areas may be more reluctant to undergo immunotherapy [3].

Although the receipt of immunotherapy was associated with area‐level SES, no marked differences in this association were observed within hospital comparisons. Our finding of lower immunotherapy receipt among patients treated at urban and academic hospitals, compared with those treated at rural and non‐academic hospitals, suggests that pharmacotherapy in an outpatient setting is more prevalent in these hospitals. This pattern was not necessarily observed in large‐sized hospitals [50]. In Japan, most first‐line pharmacotherapies, including immunotherapy and platinum‐based chemotherapy, are administered in an inpatient setting [51]. However, outpatient pharmacotherapy is gradually becoming more widespread in urban and academic hospitals, which are equipped with specialized healthcare professionals and facilities.

On the contrary, our results were generally consistent, demonstrating that patients living in socioeconomically disadvantaged areas were less likely to receive immunotherapy compared with those living in more socioeconomically advantaged areas, even after stratification by urban, non‐academic, cancer‐designated or non‐cancer‐designated, and medium hospitals. These findings suggest that patients with lower SES may be more likely to receive immunotherapy when treated at hospitals with higher immunotherapy rates for those with higher SES backgrounds. However, this could also suggest that hospitals with smaller SES‐related disparities are typically rural or academic hospitals, rather than medium and cancer‐designated hospitals. Several potential explanations exist for why rural and academic hospitals did not differ in immunotherapy rates across SES levels. In Japan, where universal healthcare coverage and free access to hospitals are provided, approximately 20% of patients with cancer are admitted to hospitals far from their residences [52]. Furthermore, a recent study has highlighted educational inequalities as significant risk factors contributing to lung cancer‐specific mortality disparities [46]. Our analysis did not include information on individual educational data; however, we hypothesize that highly educated patients from socioeconomically disadvantaged areas tend to select distant hospitals with treatment options to optimize their care. Another possible explanation is that healthcare workers' implicit biases toward patients living from low‐SES backgrounds may affect decisions regarding the administration of immunotherapy [53]. Patients from low‐SES backgrounds may receive less detailed explanations and fewer treatment recommendations, potentially resulting in missed opportunities for immunotherapy [54]. Another study highlighted that, in other areas of cancer palliative care, factors such as hospital social norms, individual clinician orientation, and unmeasurable factors at the hospital level may influence the decision to administer cancer treatments at different hospitals [55]. Regardless of cancer designation, regional and university hospitals may exhibit smaller treatment disparities based on SES, suggesting a treatment structure in which treatment decisions are less influenced by SES. However, further research is needed to confirm these hypotheses. Notably, similar trends in treatment disparities based on SES were observed at cancer‐designated hospitals, which are responsible for coordinating cooperative systems and consultations for cancer treatment. This finding aligns with a previous study conducted in Japan, which demonstrated that survival disparities based on area‐level SES remained consistent even when patients were treated at cancer‐designated hospitals [7]. These findings suggest the need for further efforts to ensure equitable access to immunotherapy, even within cancer‐designated hospitals in Japan. Further studies are necessary to examine the organizational structures of hospitals and the processes related to the administration of immunotherapy.

Our study has some limitations. First, as with any observational study, we were unable to fully account for the unmeasured confounders. Although we adjusted for patient‐level variables, unmeasured patient‐level confounding factors may have introduced bias into the results. For example, our data did not include information on oncogenic gene mutations, programmed cell death ligand 1, or the histological types of NSCLC (squamous cell carcinoma and adenocarcinoma). The absence of this information may have introduced bias in the selection of immunotherapy treatments. However, in Japanese clinical practice, most patients with NSCLC undergo testing for oncogenic mutations and programmed cell death ligand 1 expression before treatment, and treatment decisions are made according to established guidelines [29, 56]. Thus, the influence of missing this information is limited. Second, caution is warranted when interpreting the use of ADI. Area‐level SES was used to assess the receipt of immunotherapy. The observed equalities in immunotherapy for patients with stage IV NSCLC may not accurately reflect individual SES factors (e.g., personal income and education); hence, we were not able to consider the impact of individual‐level SES [4, 49]. Third, patients with low SES might be less likely to receive inpatient treatment due to financial constraints and the severity of medical conditions, which could influence the indication for first‐line pharmacotherapy. However, if this were the case, we would expect to observe widening disparities in treatment in our study. Fourth, although our study included inpatients treated with first‐line pharmacotherapy, the findings may not be generalizable to patients with stage IV NSCLC receiving other chemotherapies not included in our analysis or patients who were not hospitalized for pharmacotherapy as most first‐line chemotherapies, including immunotherapy and platinum chemotherapy, are administered in an inpatient setting [51]. Finally, due to the limited hospital information available in administrative claims data, the mechanisms underlying the disparity in the receipt of immunotherapy observed in Japan could not be identified, despite the country's universal healthcare system and caps on out‐of‐pocket payments based on household income. Although the disparity in immunotherapy use was attenuated after adjusting for hospital‐level differences, stratified analyses suggest that hospital factors, such as cancer‐designated status, do not account for the observed lower rate of immunotherapy use among patients living in socioeconomically disadvantaged areas. Future studies with more detailed information on patients' pathways for obtaining immunotherapy may help to explain the lower levels of immunotherapy among socioeconomically disadvantaged patients with stage IV NSCLC.

## 5 Conclusions

SES disparities were identified in the receipt of immunotherapy among inpatients with stage IV NSCLC in Japan, despite universal healthcare coverage and low out‐of‐pocket payments. Additionally, similar trends in immunotherapy disparities were observed at the hospital level, particularly in urban, non‐academic, and cancer‐designated hospitals. However, when hospital fixed effects were used to make comparisons within the same hospitals, SES‐based disparities in immunotherapy use were no longer observed. Identifying the underlying mechanisms that lead to these disparities may improve the quality of care, access to treatment, and outcomes for patients with NSCLC.

## Author Contributions

**Hiroe Suzuki‐Chiba:** conceptualization, methodology, software, data curation, formal analysis, project administration, writing – review and editing, writing – original draft, validation, investigation, visualization. **Atsushi Miyawaki:** conceptualization, methodology, writing – review and editing, writing – original draft, validation, formal analysis. **Taiki Hakozaki:** conceptualization, methodology, writing – review and editing, formal analysis. **Shotaro Aso:** software, data curation, writing – review and editing, resources. **Hiroki Matsui:** resources, writing – review and editing, software, data curation. **Hideo Yasunaga:** writing – review and editing, supervision, funding acquisition, project administration.

## Disclosure

This study did not involve the reproduction of material from other sources. This study was a retrospective observational study using anonymous data. Therefore, clinical trial registration was not applicable to this study.

## Ethics Statement

Ethical approval was obtained from the Institutional Review Board of the University of Tokyo (approval number: 3501‐5; May 19, 2021). This study was conducted in accordance with the ethical standards of the 1964 Declaration of Helsinki and its later amendments.

## Consent

The requirement for informed consent was waived owing to the use of anonymous data.

## Conflicts of Interest

Dr. Atsushi Miyawaki received consulting fees from M3 Inc. and Datack Inc.; lecture fees from Janssen Pharma (within the past 36 months); and grants from the Health Care Science Institute (2023–2024), the Organization of Data for Social Transformation (2023–2025), Ministry of Health Labour and Welfare (Health Labour Sciences Research Grant), and Japan Society for the Promotion of Science (24K02701) outside the submitted work. Dr. Taiki Hakozaki received lecture fees from Chugai Pharmaceutical and Ono Pharmaceutical (within the past 36 months) outside the submitted work. The rest of the authors declare that they have no conflicts of interest.

## Supporting information


Appendix S1.

Figure S1.

Figure S2.

Table S1.

Table S2.

## Data Availability

The database can only be used by licensed facilities, and the data used in this study are subject to terms of use that restrict their availability for third‐party distribution. Detailed methodologies and analyses have been included in the manuscript to facilitate reproducibility.
