# Supplementary material for: Association Between Area‐Level Socioeconomic Disadvantage and Immunotherapy in Patients With Non‐Small Cell Lung Cancer
Source: Cancer Med. 2025 Jul 10;14(13):e71038. doi: 10.1002/cam4.71038 (PMC12242713; doi:10.1002/cam4.71038)
Supplement: Supplementary file 1 — Appendix S1. [file CAM4-14-e71038-s003.docx]

**Appendix.** Methods for calculating the Japanese census-level area deprivation index

The area deprivation index (ADI) score used in this study was calculated as the weighted sum of the census variables using the following formula:

ADI_i_=k×(2.99×proportion of old couple households_i_+7.57×proportion of old single households_i_+17.4×proportion of single-mother households_i_+2.22×proportion of rental houses_i_+4.03×proportion of sales and service workers_i_+6.05×proportion of agricultural workers_i_+5.38×proportion of blue-collar workers_i_+18.3×unemployment rate_i_)

where *i* is an area index (Japanese Chocho-Aza, which is approximately equivalent to a European parish or an American block group) and k is a positive constant, which determines the percentile rank of a municipality. The value of k was initially assigned by making a synthetic estimate of the number of “poverty” households in an area.

To calculate the ADI, we used eight poverty-related census variables from the 2015 population census data.

The data were obtained from the official Japanese statistical website, as outlined in the following link:

https://www.e-stat.go.jp/stat-search/files?page=1&toukei=00200521&tstat=000001080615&metadata=1&data=1
