# Supplementary material for: Association Between Area‐Level Socioeconomic Disadvantage and Immunotherapy in Patients With Non‐Small Cell Lung Cancer
Source: Cancer Med. 2025 Jul 10;14(13):e71038. doi: 10.1002/cam4.71038 (PMC12242713; doi:10.1002/cam4.71038)
Supplement: Supplementary file 2 — Figure S1. [file CAM4-14-e71038-s005.docx]

**Figure S1**. Trends in the adjusted rate of immunotherapy receipt based on ADI quartiles in Model 1 from 2016 to 2021
