# Supplementary material for: Association Between Area‐Level Socioeconomic Disadvantage and Immunotherapy in Patients With Non‐Small Cell Lung Cancer
Source: Cancer Med. 2025 Jul 10;14(13):e71038. doi: 10.1002/cam4.71038 (PMC12242713; doi:10.1002/cam4.71038)

**Figure S2.** Adjusted rates of immunotherapy receipt stratified based on ADI deciles with equal distribution in Models 1 and 2

A. Model 1


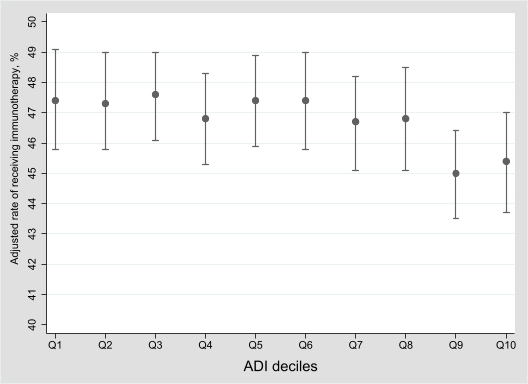


B. Model 2


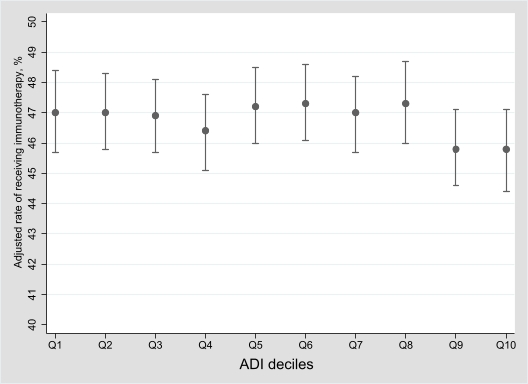

Supplement: Supplementary file 3 — Figure S2. [file CAM4-14-e71038-s004.docx]
