# Supplementary material for: Association Between Area‐Level Socioeconomic Disadvantage and Immunotherapy in Patients With Non‐Small Cell Lung Cancer
Source: Cancer Med. 2025 Jul 10;14(13):e71038. doi: 10.1002/cam4.71038 (PMC12242713; doi:10.1002/cam4.71038)
Supplement: Supplementary file 4 — Table S1. [file CAM4-14-e71038-s002.docx]

**Table S1.** Sensitivity analysis of the association between the use of immunotherapy as first-line pharmacotherapy and the ADI quartiles defined by equal participant distribution

|  |  | Model 1 | | | Model 2 | | |
| --- | --- | --- | --- | --- | --- | --- | --- |
| ADI quartile | No. of patients | Adjusted rate,  % (95% CI) | Average marginal effect, % (95% CI) | P-value | Adjusted rate,  % (95% CI) | Average marginal effect, % (95% CI) | P-value |
| Quartile 1  (least disadvantage) | 11,821 | 47.3 (46.2 to 48.5) | Reference |  | 46.9 (46.1 to 47.6) | Reference |  |
| Quartile 2 | 11,824 | 47.3 (46.2 to 48.4) | –0.1 (–1.3 to 1.2) | 0.92 | 46.9 (46.2 to 47.6) | -  ,0.0 (–1.2 to 1.1) | 0.96 |
| Quartile 3 | 11,824 | 46.9 (45.7 to 48.1) | –0.5 (–1.8 to 0.92) | 0.05 | 47.1 (46.3 to 47.8) | ,0.2 (–1.1 to 1.4) | 0.79 |
| Quartile 4  (most disadvantage) | 11,822 | 45.6 (44.4 to 46.8) | –1.7 (–3.2 to –0.3) | 0.02 | 46.2 (45.5 to 47.1) | –0.6 (–1.9 to 0.7) | 0.15 |

Adjustments were made for sex, age, body mass index, smoking index, Charlson Comorbidity Index score, interstitial lung disease, connective tissue disease, Barthel Index score, and rural index, matched by patient residential area, emergency admission, and fiscal year of admission (Model 1). To account for the potential correlation among patients treated at the same hospital, hospital fixed effects were additionally included in the model (Model 2).

Abbreviations: ADI, area deprivation index; CI, confidence interval
